# Leprosy incidence and risk estimates in a 33-year contact cohort of leprosy patients

**DOI:** 10.1038/s41598-021-81643-4

**Published:** 2021-01-21

**Authors:** Mariana Andrea Hacker, Anna Maria Sales, Nádia Cristina Duppre, Euzenir Nunes Sarno, Milton Ozório Moraes

**Affiliations:** Leprosy Laboratory, Oswaldo Cruz Institute, Oswaldo Cruz Foundation, Avenida Brasil 4365, Manguinhos, Rio de Janeiro, RJ 21040-360 Brazil

**Keywords:** Epidemiology, Infectious diseases

## Abstract

Reduction in incidence has been associated with the introduction of novel approaches, like chemo/immune-prophylaxis. Incidence determined through follow-up cohort studies can evaluate the implementation of these innovative policies towards control and prevention. We have assessed the incidence in our contacts cohort over past 33 years, considering the effect of demographic and clinical variables. Survival analysis was used to estimate the risk of leprosy. A total of 9024 contacts were evaluated, of which 192 developed leprosy, resulting in an overall incidence of 1.4/1000 person-years. The multivariate analysis showed that the major risk factors were (i) contact from MB index cases and (ii) consanguinity (iii) intra household contact. Lower risk was detected for contacts with BCG scar who were revaccinated. There was a significant decrease in accumulated risk between the 2011–2019 period compared with 1987, probably linked to the improvement in laboratory tools to monitor contacts, thereby providing early diagnosis of contacts at intake and reduction of transmission. Our findings suggest that a combination of contact surveillance and tracing, adequate neurodermatological examination, and availability of molecular tools is highly effective in supporting early diagnosis, while a second dose of the BCG vaccination can exert extra protection.

## Introduction

Leprosy is a neglected disease exhibiting a plateau of 200,000 new cases worldwide each year, despite the efficacy of multidrug therapy that has cured millions of patients over the past 30 years. Brazil, India, and Indonesia concentrate the highest number of new cases. Delayed diagnosis is responsible for active transmission before treatment^1–3^. According to the World Health Organization (WHO), the number of cases registered in 2018 was 208,619 with a prevalence rate of 0.2/10,000^4^. In Brazil specifically, 28,660 new cases were registered in 2018. Studies show that although there is a downward trend in the detection rate of new cases in the country over the past 10 years^5^, some changes in the epidemiological features, such as increased incidence among older people developing leprosy, were observed^6,7^, while some regions still show stagnant or increasing rates^8^. In particular, leprosy persists in the north and northeast regions as a critical public health problem^9,10^, demonstrating the heterogeneity of the epidemic in Brazil.

The epidemiological situation of leprosy can be monitored by several indicators, with the incidence being the most relevant epidemiological measure of the disease burden. The household or neighbor and social contacts of leprosy patients constitute the group at greatest risk of developing the disease, thus being the targeted population for leprosy control^11^. In Brazil, few studies have measured incidence in contacts from a follow-up cohort^12–15^. The factors associated with disease outcome among contacts include consanguinity, proximity, bacterial index/clinical form of the index case, age, and sex^2,12–18^.

Contact tracing and geographic information systems studies reveal a concentration of new patients among household and social contacts, either children or adults, of previous patients highlighting the importance of developing differentiated actions for surveillance of this group^19–21^. Simulation studies^22^ and systematic reviews^23^ suggest that the control of leprosy could be achieved faster through household contact tracing associated with new interventions such as chemo or immunoprophylactic approaches^17,18,24^. Indeed, immunoprophylaxis and contact surveillance has an impact on outcome^17,24^. Nevertheless, combination of a single dose rifampicin after BCG vaccination did not provide extra protection as compared to second dose of BCG alone^25^. Importantly, different studies demonstrate that contact surveillance is less than ideal and still encounters operational problems, particularly in the context of social vulnerability of the targeted populations, which is generally where leprosy persists in high endemicity^26,27^.

The analysis of the incidence of leprosy among contacts may contribute to the evaluation and planning of control and prevention measures. The FIOCRUZ clinic is a national reference center for the diagnosis and treatment of leprosy patients and contact surveillance, and has been following a cohort of contacts since 1986. The surveillance of contacts is carried out through dermato-neurological examination, although since 2011 qPCR has also been used as a routine screening tool for follow-up in this group of individuals. The aim of this study was to evaluate the incidence of leprosy in the cohort of contacts considering the year of diagnosis of the index case as a cohort year. The effects of the studied variables on disease outcome were used to estimate the risk also including two periods (1987–2010 and 2011–2018) analyzed as variable divided as based on the qPCR introduction as routine screening tool of contact.

## Results

In the analyzed period (1987–2018), a total of 9024 contacts were evaluated, of which 192 developed leprosy, resulting in an incidence of 1.4/1000 person-years. The epidemiological profile of the contact cohort is given in Table 1. The average age of the contacts was 27 years old (standard deviation 18.6) and the median was 24, with approximately 58% of the cohort being women. Most contacts had a BCG scar (71.3%) and 75.8% took a dose of the vaccine at examination, either first vaccination or a booster/revaccination. Most of them were consanguineous contacts from multibacillary patients.

**Table 1:** Epidemiological profile of the cohort of contacts at the FIOCRUZ clinic, 1987–2018.

Variables	N (%^a^)
**Sex**	
Male	3761 (42.2)
Female	5154 (57.8)
**BCG vaccination**	
None	305 (4.2)
BCG scar^b^	1468 (20.0)
Revaccination^c^ (BCG scar + new dose)	3756 (51.3)
BCG applied at examination^d^	1793 (24.5)
**Consanguinity**	
Yes	6389 (73.2)
No	2345 (26.8)
**Type of household contact**	
Intra-household contact	4667 (54.4)
Extra-household contact	3916 (45.6)
**Index case operational classification**	
PB (Paucibacillary)	2927 (33.7)
MB (Multibacillary)	5762 (66.3)
	**Average (standard deviation)**
Age	27 (18.4)
Follow-up time (years)	15(9.2)

^a^Proportions were calculated for valid data, excluding the respective missing data: n = 109 (1.2%) for sex, 1702 (18.9%) for BCG vaccination, 290 (3.2%) for consanguinity, 441 (4.9%) for intra/extra household and 335 (3.7%) for operational classification.

^b^Only BCG scar.

^c^Scar + new BCG vaccine dose applied at first examination.

^d^BCG applied at first examination without previous scar.

**Figure 1:**
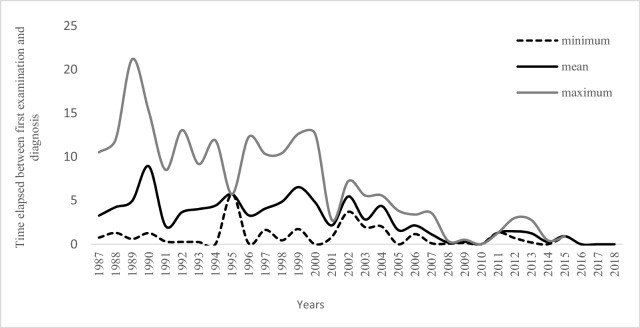
Time in years for disease outcome in the contacts cohort, according to year of the first examination. Cohort of contacts at the FIOCRUZ clinic, 1987–2018. Dotted line-minimum; black mean; gray-maximum.

The average follow-up time for contacts was 15 years. In Fig. 1, the average time of disease outcome (time elapsed between first examination and diagnosis) decreased over the years analyzed in the cohort.

In the bivariate analysis, there was a statistically significant difference in the accumulated risk between the two analyzed periods, indicating greater risk in the first period. As expected, significant differences were observed in the risk function according to the presence of a BCG scar, new BCG vaccination, consanguinity, and operational classification of the index case. The cumulative risk did not show significant differences according to the age and sex of the contact in this analysis (Table 2).

**Table 2:** Bivariate and multivariate analysis of the risk of disease outcome among contacts using Cox regression model.

Variables	Unadjusted HR (95% CI)	Adjusted HR (95% CI)
Period 1987–2010 (ref: 2011–2018)	2.57 (1.51–4.37)	3.98 (1.84–88.62)
Sex female (ref:Male)	1.18 (0.88–1.58)	1.41 (0.987–203)
Age	1.01 (0.99–1.01)	0.99 (0.99–1.01)
MB index case (ref:PB)	3.97 (2.53–6.26)	4.05 (2.32–7.07)
Consanguinity (ref:No)	1.32 (0.93–1.87)	1.62 (1.03–2.55)
Intra-household contact (ref: extra-household contact)	1.44(1.07–1.94)	1.45(1.06–2.08)
**BCG vaccine**		
BCG scar	0.24 (0.14–0.42)	0.25 (0.14–0.47)
Revaccination (BCG scar + new dose)	0.14 (0.09–0.24)	0.13 (0.08–0.23)
BCG applied at examination	0.22 (0.13–0.37)	0.22 (0.12–0.38)

Cohort of contacts at the FIOCRUZ clinic, 1987–2018.

**Figure 2:**
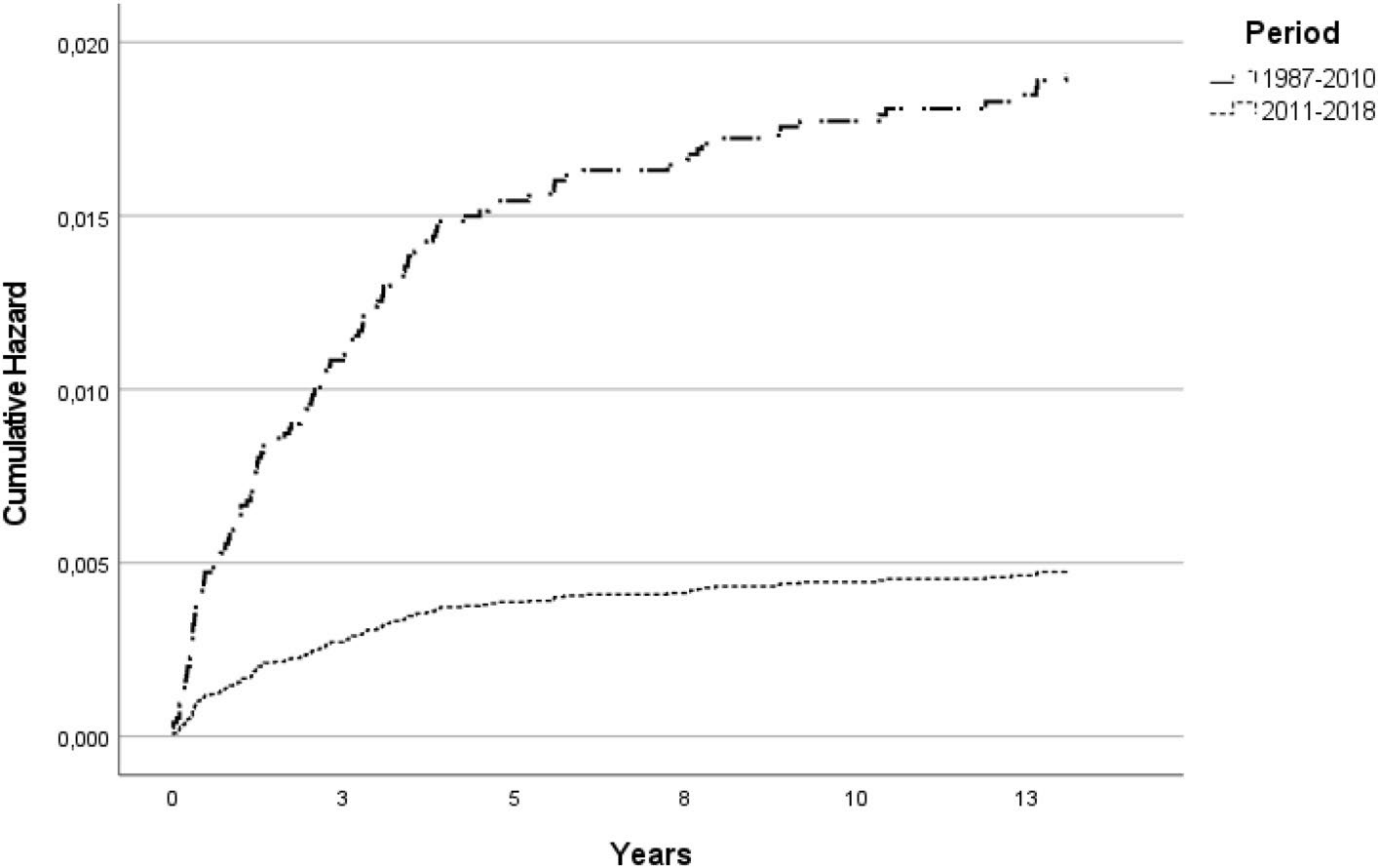
Risk function for the periods (1987–2010 and 2011–2018) according to Cox regression model adjusted for age, sex, BCG (scar and vaccination), consanguinity, type of household contact and classification form of index case. Cohort of contacts at the FIOCRUZ clinic, 1987–2018.

The multivariate analysis in our curated dataset of more than 9000 contacts showed a higher risk for the contacts of MB index cases (HR 4.05, CI 2.32–7.07) and if they were consanguineous (HR 1.62, CI 1.03–2.55) and intra-household contact (HR 1.45, CI 1.06–2.08). Protection of 75% was detected for contacts exhibiting a BCG scar, 78% for those without a scar but who had their first BCG vaccination at intake, and 87% for those revaccinated at examination (new dose + BCG scar). Sex and age did not show a statistically significant difference (Table 2). The comparison between the two follow-up periods (1987–2010 and 2011–2018) as a variable using Cox regression showed that, with adjustments using other clinical and epidemiological variables associated with risk, the difference in accumulated risk remains significant between the two periods (HR 3.98, CI 1.84–8.62). It was observed that after controlling for the distribution of the other variables in both cohort periods, the difference between the two periods became more pronounced (Table 2). Then, the estimated curves for both periods considering the other explanatory variables of the model were generated (Fig. 2), showing a higher and growing risk for the first period over the years of follow-up. In the second period, the risk, in addition to being lower, does not show an upward trend over the years (Fig. 2).

## Discussion

Here, we present data from a long and extensive contact cohort. The combination of the high number of contacts and the long follow-up period, enabled a reduction in the risk of disease outcome after 2010 to be identified. The second period analyzed (2011–2019) was 9 years, which was a shorter follow-up period than the first (1987–2010) and could be considered limited. Nevertheless, the statistical model used made it possible to compare the risk of disease outcome between the two analyzed periods considering the difference of follow-up time and regardless of other factors, such as the clinical form of the index case, consanguinity, presence of a BCG scar, and a new dose of BCG vaccination. The main difference between the two periods is the use of qPCR as a routine tool to screen and monitor contacts. We observed that indeed, in our experience, qPCR was very important to help dermatologists in defining new cases among contacts that, at intake, already had a lesion. Our clinic routinely performs diagnosis of difficult cases aided by the use of qPCR^28^. The availability of this method provides the clinicians the possibility to interrogate leprosy when histopathological analysis of prevalent contacts with smaller lesions examined at intake are assumed equivocal or inconclusive^13,28^. In Manta et al.^13^ we followed up 980 contacts for at least 3 years after diagnosis of the index cases. In this group, 25 contacts showed a leprosy-like skin lesion. A total of 8 of them were diagnosed as leprosy and 50% had a positive qPCR result. On the other hand, the 17 patients diagnosed as other dermatological disease, only one (6%), were qPCR positive. Thus, the qPCR influenced a clinical decision towards leprosy diagnosis in this group of co-prevalent cases, when contacts already have a lesion. It is important to notice that these co-prevalent cases were all PGL-I and slit skin smears from ears lobes negative. Therefore, it is likely that the use of qPCR as a support for clinicians could impact the differences in these two periods.

Nevertheless, since we introduced qPCR as a new approach to support early diagnosis during screening of contacts with skin or neurological lesions it is still necessary for independent replication of our findings. The routine practice after introduction of qPCR was effective in our clinic. Indeed, a systematic review and meta-analysis indicates that PCR has good accuracy, although studies are highly heterogeneous suggesting that large multicentric studies using good manufacturing practices products for diagnostic purposes should be used for validation of the present data^29^.

Our previous studies conducted in an interim analysis of this cohort showed that household exposure and index case’s elevated bacterial load were associated with leprosy^12^. Also, the effectiveness of protective responses after BCG vaccination^24^ and the kinship susceptibility to the disease, suggesting that both genetic susceptibility and physical exposure play an important role in the epidemiology of leprosy^30^. Gender was not found to be associated with leprosy in this analysis but being male have been associated with leprosy previously^2^.

Here, we showed the protective effect of BCG vaccination (previous vaccination as measured by the presence of a scar), revaccination or recent primary dose, independently of others factors like clinical form of the index case and consanguinity. Meta-analysis studies showed that an additional dose of BCG was more protective in the prevention of leprosy, suggesting an important strategy in areas where leprosy continues to be a public-health problem^31–33^. The protection is higher in household contacts of leprosy patients, but evidence for the benefit of re-vaccination is conflicting^31^. Previous literature showed the efficacy of BCG protection on leprosy according to age at vaccination, clinical form and number of doses^24,33^.

However, the overall analysis of incidence indicates a decrease in the past 30 years and several other factors are contributing to this reduction. Leprosy is strongly associated with the social context, where not only individual, but also collective aspects are associated with disease^34^. In the past decade, especially after 2004, several social protection programs were integrated, improved and expanded, such as Bolsa Família (from Portuguese, Family Allowance Program, BFP), a conditional cash transfer program to the most vulnerable populations, reaching millions of Brazilians^35,36^. In this regard, a large Brazilian study using a database of 100 million people and correlated socioeconomic data with leprosy cases and found that lower levels of income, education, and factors related to poor housing conditions are associated with an increased incidence of the disease^3,14^. It is likely that our cohort was also impacted by social protection programs in Brazil contributing to the reduction in disease development that was most detected in period of the past 9 years.

Another interesting finding of this current analysis is the reduction in the time of disease outcome. We observed earlier diagnosis being achieved over the years, possibly due to the evolution of techniques to support diagnosis and professional training, as well as advances in epidemiological surveillance practices, including health promotion and education. However, FIOCRUZ is a national reference center, where a multidisciplinary team acts in an integrated manner with adequate infrastructure. Therefore, the findings may not represent the reality existing in other health care units in Brazil^37^. A recent study described the trend in detection rates in Brazil, from 1990 to 2016, where it is possible to observe a significant trend of decline in the detection rate in the country, especially in the South and Southeast^6^.

Estimating the incidence of leprosy is conditioned by several clinical and operational aspects of the disease and the mechanisms of detection and surveillance in different social contexts and infrastructure conditions of health services. More recently, other parameters such as “years of life adjusted for quality” (better known by the English acronym QUALYs) have been used as a measure for epidemiological assessments^38^. However, these measures are often performed assuming that incidence is equal to prevalence, due to the impossibility of estimating incidence^39^. In leprosy, the detection rate is used as a proxy for incidence, given the difficulty of early diagnosis of the new case and the long incubation period of the disease. However, the cohort-based incidence estimate allows for a more accurate assessment of the trends and magnitude of the disease, in order to support epidemiological surveillance and disease control actions. The present study made it possible to assess the incidence of leprosy among contacts, due to the cohort established in surveillance. The initial examination performed at the time of the diagnosis of the index case identified healthy individuals and accompanied them for years in search of early diagnosis.

We observed earlier diagnosis being achieved over the years, possibly due to the evolution of techniques to support diagnosis, as well as advances in epidemiological surveillance and health education.

The use of diagnostic tests has been largely discussed. A meta-analysis study regarding the accuracy of ELISAs in detecting antibodies against mycobacterium leprae concluded that traditional ELISAs have good accuracy in detecting MB leprosy and poor accuracy in detecting PB leprosy^40^. Another meta-analysis evaluating diagnostic accuracy of tests concluded that although the test accuracy looks reasonable, the studies suffered from heterogeneity and low methodological quality^41^.

In summary, due to the long incubation period to disease outcome, changes in the detection rate occur slowly and are related to factors such as coverage of BCG vaccination, socioeconomic development, and contact tracing, among other surveillance actions of the control programs^2,3,17–19^. In light of our findings, it is likely that a combination of contact surveillance and tracing, adequate clinical neuro-dermatological examination, support of molecular tools for diagnosis, and a new dose of BCG vaccination could be highly effective supporting early diagnosis and decreasing detection overtime^15,42^. Perhaps, addition of chemoprophylaxis prior to BCG vaccination, which is currently being tested in our clinic in a double-blind placebo study^43^, could have further hampered transmission and impacted disease outcome.

## Methods

### Contact cohort

The contact cohort at the FIOCRUZ clinic is made up of the contacts of all leprosy patients diagnosed in the period from 1987 to 2018. People assisted at the clinic consist mostly of individuals from Rio de Janeiro and the metropolitan area, and to a lesser extent, from other municipalities in the state. Patient demand is made up of referrals from public or private health services as well as spontaneous demand.

All socio-demographic, clinical, and epidemiological information and data from the dermato-neurological examination are continually inserted in the clinic database. The contacts included in this study come from index cases registered at the clinic and only those who were healthy at intake were considered for this analysis. Contacts were examined at the time of diagnosis of the index case. Healthy contacts are taught to return to the clinic if any signs or symptoms of the disease appear.

After clinical, histopathological, and bacteriological confirmation of leprosy, the cases are classified according to their clinical form, as described by Ridley and Jopling^44^, as well as the treatment and mode of transmission. All patients are also graded according to the pauci and multibacillary classification and treated according to the WHO and Ministry of Health regulations.

In order to identify contacts from the cohort that could have been diagnosed in other health units, a search of the contacts was carried out in the National System of Notifiable Diseases (SINAN) among the cases registered in the State of Rio de Janeiro (data available from 2001 to 2018), using a probabilistic method through the Reclink program^45^.

### Statistical analysis

Incidence of leprosy was calculated among contacts over time according to the year of diagnosis of the index case up to the year of follow-up of the cohort. The follow-up time for each individual was calculated from the time elapsed between the date of diagnosis of the index case and the date of disease outcome for the contact or censorship of the contact's follow-up. This number was converted into person-years for the analysis. The contacts were aggregated by year of diagnosis of the index case, forming different cohorts according to periods for comparison. For the purpose of calculating the accumulated risk, two periods were considered for the analysis, from 1987 to 2010 and from 2011 to 2018.

The analysis was performed considering the variables of interest to the study: operational classification of the index case, consanguinity, intra/extra household contact, BCG scar (previous vaccination) and BCG vaccination (shot after intake as leprosy contact), sex and age. Survival analysis was used to estimate the accumulated risk curves. Cox regression was used to estimate the risk of disease in both periods of the study, considering the effect of all other variables simultaneously. Statistical significance level was established at 5%. SPSS (Statistical Package for Social Science) was used for statistical analysis.

### Ethics declaration

After receiving educational information about leprosy, all adult participants and the parents or guardians of the child participants provided informed consent. A medical history for each contact was taken from routine care medical records. Data collection, management, and analysis were performed by the study coordinators, and confidentiality was maintained throughout the research. The present study, including the use of patient records, was approved by the Research Ethics Committee of the National School of Public Health (Document #113/06).
