# Author Correction: Leprosy incidence and risk estimates in a 33-year contact cohort of leprosy patients

**DOI:** 10.1038/s41598-021-88096-9

**Published:** 2021-04-20

**Authors:** Mariana Andrea Hacker, Anna Maria Sales, Nádia Cristina Duppre, Euzenir Nunes Sarno, Milton Ozório Moraes

**Affiliations:** Leprosy Laboratory, Oswaldo Cruz Institute, Oswaldo Cruz Foundation, Avenida Brasil 4365, Manguinhos, Rio de Janeiro, RJ 21040-360 Brazil

Correction to: *Scientific Reports* https://doi.org/10.1038/s41598-021-81643-4, published online 21 January 2021

The original version of this Article contained errors in Table 2, where the Unadjusted HR values were incorrect for “BCG scar”, “Revaccination (BCG scar + new dose)” and “BCG applied at examination”. The correct and incorrect values appear below.

Incorrect:

**Table:** 

Variables	Unadjusted HR (95% CI)
**BCG vaccine**	
BCG scar	3.39 (2.39–4.81)
Revaccination (BCG scar + new dose)	0.81 (0.59–1.10)
BCG applied at examination	0.49 (0.38–0.63)

Correct:

**Table:** 

Variables	Unadjusted HR (95% CI)
**BCG vaccine**	
BCG scar	0.24 (0.14–0.42)
Revaccination (BCG scar + new dose)	0.14 (0.09–0.24)
BCG applied at examination	0.22 (0.13–0.37)

The original Article has been corrected.

